# Communication strategies used by adolescents with autism spectrum disorder and health professionals during treatment

**DOI:** 10.4102/ajod.v11i0.811

**Published:** 2022-03-31

**Authors:** Monica Araujo, Munyane Mophosho, Sharon Moonsamy

**Affiliations:** 1 Department of Speech Pathology and Audiology, Faculty of Humanities, University of the Witwatersrand, Johannesburg, South Africa; 2 Department of Speech Therapy and Audiology, Faculty of Humanities, University of the Witwatersrand, Johannesburg, South Africa

**Keywords:** ASD, adolescents, health professionals, interactional styles, communication breakdowns, effective interactional strategies

## Abstract

**Background:**

Autism spectrum disorder (ASD) is a developmental disorder, which affects social communicative capabilities. The research study has shown that ASD studies are focused on young children, excluding adolescents and adults: and it is understudied in the context of South Africa.

**Objective:**

This study examined the interactional communication strategies of adolescents with ASD and health professionals during different treatment consultations to identify the interactional styles and communication strategies utilised by adolescents with ASD and their respective healthcare professionals in a variety of scenarios in order to generate management strategies for future healthcare professional communication training.

**Method:**

A multi-case study design with a qualitative research approach has been used. Four adolescents with a moderate form of ASD and four health practitioners were interviewed. Participants were chosen by purpose and snowball sampling. Semi-structured, open-ended interviews were used for health professionals to collect information on the various interaction types and communication methods used, as well as their interpretations of these methods. Conversely, adapted face-to-face interviews were used to collect similar knowledge from adolescents themselves. The findings were qualitatively analysed on a case-by-case and cross-case basis by thematic analysis techniques.

**Results:**

The findings indicated that ASD adolescents have interaction types that influence intervention to various degrees. In comparison to motor therapies such as occupational therapy and physiotherapy, interaction types have a greater impact on psychiatry and psychology, which depend mainly on verbal communication. Intuitively, to promote contact with these teens, all health practitioners changed their own interaction styles. They used techniques of clarification and repair. The therapists shared the intention to learn a range of successful ways to strengthen future experiences with ASD between themselves and adolescents.

**Conclusion:**

The findings indicate that practitioners can benefit from altering their interaction styles, and that approaches for promoting successful interactions and in establishing rapport could be shared with other professionals in the future.

## Introduction

Individuals with autism spectrum disorder (ASD) exhibit both social, interactional and communication breakdowns (Mody & Belliveau 2013). As a result of the complex communication needs of these individuals, many of them require social skills training and effective communication support through the use of beneficial communication strategies and possibly even augmentative and alternative communication (AAC) (Trembath et al. 2014). Moreover, many of these individuals require specialised and individualised care (Sicile-Kira & Sicile-Kira 2012), and as a result, they normally consult a combination of health professionals who take on different roles and responsibilities in addressing the interactional breakdowns experienced by individuals with ASD. These professionals include physicians, psychologists, speech language therapists (SLTs), physical therapists and occupational therapists (OTs) (Sicile-Kira & Sicile-Kira 2012). Engagements between the healthcare practitioners and the individual with ASD hinge on their communication interactions. Buller and Buller (1987), indicated that patients who believe that their physicians’ communication style is relationship-oriented are more likely to be satisfied with their medical care. Many studies on communication and interactional styles between patients and healthcare practitioners often reflect interactions amongst medical doctors and their patients, as well as between nurses and their patients (Levin 2005; Macdonald, Carnevale & Razack 2007). Limited evidence of published studies between healthcare professions (SLTs, OTs, physiotherapists) and individuals with ASD have been observed. Hence, this study examined the interactional styles and communication strategies used by adolescents with ASD and their health professionals, as well as perspectives of their parents, within Gauteng, South Africa. For the purpose of this article, the interactional styles and communication strategies used by adolescents with ASD and their health professionals are highlighted.

## Literature review

In South Africa, the prevalence of ASD is still unknown. According to Springer et al. (2013), there were over 270 000 individuals with ASD in South Africa and a predicted 5000 new cases of ASD each year, with this number increasing yearly (Springer et al. 2013). Despite the growing rate of ASD, local and international research on autism lags behind that of other medical conditions and psychiatric disorders (Thurm & Swedo 2012). In addition, the research on ASD that exists focuses mainly on young children. Thus, there remains substantial gap in the literature on the communication, participation and overall life challenges experienced by individuals with ASD beyond childhood, including adolescents and adults (Howlin & Taylor 2015; Miller et al. 2014).

The popular areas of focus in ASD research have explored the effects of ASD on the individual’s activities of daily living, well-being, and aspects of friendship, loneliness, stigma, general social challenges, independence, parent perceptions and distress. However, fewer studies are currently available on the interactional styles and communication breakdowns experienced by adolescents with ASD in their therapy interactions with their health professionals. As individuals with ASD interact with multiple health professionals throughout their lifetime, understanding the common interactional styles and breakdowns would have implications for the success of various interventions. Thus, communication styles and interactions between health professionals and patients are explored so that communication breakdowns can be identified, as communication has implications for health behaviours.

When young typically developing (TD) children’s communication breaks down, they create repair strategies. It’s an important aspect of the language learning process. Repair mechanisms are still in the early stages of development and begins without instruction at a young age. Children with sentences with mean length utterance (MLU) of 1.5 to 2.9 repeat or revise their message in response to requests for clarification. When prompted to clarify, 4-year-olds respond positively and are sensitive to signs that the clarification satisfied the listener. Children from 34 to 67 months react to various forms of clarification requests at least 80% of the time. So language learners who are relatively inexperienced are sensitive to criticism that their messages are not being comprehended. They have the ability to change in order to be understood during interactions (Anderson 2013). However, one of the most challenging problems for children with communication challenges such as ASD is learning how to communicate appropriately and to repair communication breakdowns. Their conversation is comparable to that of younger TD children whose language is in the process of developing (e.g. reach for food or vocalise to gain attention).

These alternative techniques are usually misconstrued by others who hear them; therefore, providing children with tools to repair misunderstandings is critical if they are to effectively influence the behaviour of others. When the first communication attempt fails, the ability to continue communicating whilst also changing, repeating or revising a signal may be defined as a communicative repair (Wetherby & Prizant 1993). Brady et al. (2002), Halle et al. (2004), Keen (2003) and Wetherby et al. (1998) have suggested a link between communication breakdowns, repairs and problem behaviour in children. Keen (2003) proposed two theories on behaviours in the context of broken communication, that is, a type of protest or frustration after a failed attempt to express a need. Scudder and Tremain (1992) discovered that children with cognitive delays become more frustrated as the request sequence advanced. According to Keen, a second explanation for challenging behaviour during breakdowns is that it may be a form of repair strategy. Halle et al. (2004) claimed that behaviourally, repairs happening in response to breakdowns are analogous to behaviour extinction. If the ‘new’ repair topography succeeds (e.g. the child gets the desired object), the previously reinforced topography weakens and the more problematic behaviour strengthens. If the problem conduct is reinforced regularly, it may become an initial request topography as well. Communication repair strategies are therefore important in client and practitioner interactions.

The type of communication between practitioner and patient or client has been found to influence health behaviour of individuals. Patients and practitioners frequently have divergent viewpoints on therapeutic encounters, with patients placing a value on the practitioner’s ability to comprehend and accept their status. Whilst patients’ perceptions are critical to clinical outcome, they may not accurately reflect all relevant aspects of treatment delivery (Larsson et al. 2014), as a result, a thorough analysis of practitioner and patient reflections, is deemed important. According to research on doctor–patient communication, poor communication contributes to poor health outcomes, poor patient compliance and poor patient commitment to the intervention and treatment regimens (Levin 2005; Macdonald et al. 2007). Despite some research investigating nurses and individuals with ASD, research has not focused on interactional styles and breakdowns between adolescents with ASD and other health professionals. This study is, therefore, foundational in identifying communication breakdowns between adolescents with ASD and their respective healthcare professionals.

Morrison et al. (2020) found that when comparing TD adults and adults with ASD, the adults with ASD were more awkward, less attractive and socially less warm. In addition, TD adults expressed an interest in future interactions with other TD adults, whilst adults with ASD preferred interactions with adults with ASD rather than with TD persons. These findings suggest that when adults with ASD are matched with other persons with ASD, social connection may develop. Furthermore, theory of mind (ToM), which is the ability to see another’s perspective is important in communication interactions (Levey 2019), especially in communication interaction of individuals with ASD.

Research has argued that ASD is characterised by ToM deficiencies, with empirical evidence to support (Baron-Cohen 1995). Impairment in ToM results in a number of challenges in social interaction and communication. In a medical consultation, the healthcare professional must be able to understand their patient’s thinking, as well as communicate, effectively. This is significant because the patient may, on occasion, express the genuine issues that the doctor needs to know about, during the consultation, whether purposefully or accidentally.

Thus, an inability to gauge a person’s thinking is socially hampering. When conversational partners have different social norms and expectations (as is typically the case for persons with ASD); there is a tendency for a ‘disjuncture in reciprocity’, or a lack of empathy, according to Milton (2012:883). He called this a ‘double empathy problem’, in which people with ASD face challenges in social connection because of bidirectional failures in understanding what is the other person’s thinking. Recent data reveal that people with ASD (Edey et al. 2016) demonstrate thinking patterns that are difficult for their communication partners to perceive. These communication behaviours of individuals with ASD will influence their interventions, thereby impacting their quality of life.

We argue that it is pivotal to gain awareness of the possible interactional styles and communication breakdowns that exist in sessions between health professionals and adolescents with ASD. The findings of this study should make a relevant contribution to the knowledge on ASD in the South African context and globally. This knowledge will provide insight into (1) how to navigate communication barriers between individuals with ASD and health professionals, (2) emphasising the overlooked voice of adolescents with ASD in the context of ASD research, (3) developing new strategies for health professionals, which enhance interactions with their patients with ASD and those close to them (Cesa & Mota 2017). The necessity for training of health professionals will also be identified, regarding effective interactional and communication strategies that can be utilised when consulting adolescents with ASD. This study, thus, provides an analysis of communication and interactional styles during consultations, as reported by healthcare professionals and adolescents with ASD.

## Main aim of study

This study aimed to determine the interactional styles and communication strategies used by adolescents with ASD and their respective health professionals in various contexts in order to develop management strategies when providing future training to related health professionals.

### Objectives of the study

- To determine the interactional styles and communication breakdowns of adolescents with ASD in general life, as stated by the adolescents (and their parents).
- To establish the interactional styles and communication breakdowns of adolescents with ASD when communicating with their respective health professionals.
- To determine the interactional styles and communication breakdowns experienced by health professionals when communicating with adolescent clients with ASD.

## Research question

What are the interactional styles and communication strategies used by adolescents with ASD and their respective health professionals?

### Research paradigm

The interpretive paradigm was selected for this study, as the primary goal was to interpret the subjective understanding of the participants and to describe the participants’ real-life experiences without imposing a theoretical standpoint. Thanh and Thanh (2015) indicated that health professionals attempt to assign meaning to their experience of interacting with an adolescent with ASD based on their personal beliefs and understandings. The interpretive paradigm was, therefore, selected as appropriate to avoid such researcher bias.

## Research design

A qualitative, multiple-case study research design was implemented with four adolescents on the autism spectrum and the health professional that they currently see or have seen within the past 6 months. The multiple-case study design provides the reader with a vivid experience, as opposed to other stringent analytical research methods (Zach 2006). In addition, qualitative research offers a more detailed exploration of the topic (Creswell & Creswell 2012), as there are limited research studies regarding adolescents with ASD and their interactions with health professionals. Triangulation was also utilised in this study to capture diverse dimensions of the same phenomena and to foster a widespread understanding of these phenomena (Carter et al. 2014). The use of multiple data sources fosters a wider understanding of the research subject and increases the confidence in results obtained.

### Researcher reflexivity

The researcher practised reflexivity. Her observations, thoughts and interpretations were observed in a journal after each interview before the data were analysed. Patnaik (2013) stated that reflexivity keeps biases, attitudes, values and views of the world in check, thereby reducing potential influence in the analysis of data.

### Context

The research study was conducted at two schools. School A provides individuals on the autism spectrum, with a safe environment for learning, whilst being cognisant of their distinctive breakdowns; assisting them to gain a specific level of independence and to develop essential life skills. School B is an inclusive and encouraging environment that provides support for remedial and special needs students. Within School B, there is a team of support staff, professional educators and therapists who work in a holistic manner to provide differentiated learning opportunities, suitable for their students. Both schools include adolescents on the autism spectrum, which is the target population for this research study. The students saw their health professionals, privately and on site in School B.

## Data collection methods

Semi-structured, open-ended interviews were conducted with the health professionals at the school (adolescents 1 and 4) and at their respective practices (adolescents 2 and 3). The researcher also attempted to interview the health professionals before the adolescents themselves; however, this was not always possible. Interviewing the health professionals before the adolescents would have allowed the researcher to gain more information on the adolescents, as well as how they communicate with the health professional and what breakdowns are experienced in the interactions.

The adolescents with ASD were interviewed at their respective schools. However, because of their social interaction and communication challenges, certain adjustments and support systems often needed to be implemented when conducting the interviews with some of the adolescents. The adaptations included rephrasing or simplifying of specific questions, asking close-ended questions and using an adapted version of Talking Mats (a visual tool where picture symbols related to the topic of discussion are placed on a mat) to supplement their responses (Murphy et al. 2010). The researcher adapted the communication style and used questions that were concrete as opposed to abstract, to facilitate ease of processing for individuals with ASD. Open-ended questions were used when the participants were able to cope with them.

### Credibility

The following strategies were incorporated to increase credibility, trustworthiness and rigour of the findings (Noble & Smith 2015): accounting for personal biases, which could have impacted the findings, careful record keeping, signifying a clear decision path, and guaranteeing that interpretations of data are both reliable and transparent and inclusion of thick and rich verbatim explanations of participants’ accounts, to support results.

## Description of instruments

The response format for this study was face-to-face interviews, as this allowed the interviewer to pose questions to the participants and encourage open discussion, whilst providing prompts where necessary (McMillan & Schumacher 2010). Each of the interviews first included questions to attain each participant’s demographic information. Some of the topics of discussion that were used with adolescents and the health professionals are included here.

## Interview of adolescents

- Feelings towards their respective health professional, attending therapy sessions or consultations, and the activities in therapy, as well as communication and interaction with health professionals.
- Perceived breakdowns in communicating or interacting with health professionals.
- Whether or not the health professional communicates effectively with the adolescent with ASD.

The duration of the interviews with the adolescents with ASD varied and depended on their attention, focus and motivation. The interviews were also audio recorded, with the permission of all participants involved.

## Interview of health professionals

- Describe the interactional style experienced with the adolescent during therapy.
- Describe your communication with him or her.
- Describe the challenges that you experience when communicating or interacting with this adolescent.
- How do you manage or overcome these challenges?

### Participants

The demographics of the participants are presented in Table 1.

**TABLE 1:** Demographics of the participants.

Participant	Adolescent	Health professional interviewed
P1	**Age:** 14 years. **Diagnosis:** Mild autism, according to the Childhood Autism Rating Scale (CARS) assessment. **School:** School A **Length of attendance to therapy:** Attended physiotherapy for approximately 1 year.	Neuro-physiotherapist
P2	**Age:** 11 years **Diagnosis:** Low support autism according to the ADOS assessment. **School:** School B **Length of attendance to therapy:** Has been attending occupational therapy for 4 years.	Occupational therapist
P3	**Age:** 12 years **Diagnosis:** Asperger’s syndrome. **School:** School B **Length of attendance to therapy:** Has been attending psychology for 6 weeks.	Psychologist
P4	**Age:** 17 years **Diagnosis:** Asperger’s syndrome. **School:** School B **Length of attendance to appointments:** Has been to consultations with psychiatrist for 6 years (adolescent goes every 3 months).	Psychiatrist

### Data analysis

According to Braun and Clarke (2013), six phases of thematic analyses were implemented when analysing the data obtained from the various interviews within each case and across the cases. The broad themes reflected the following: interactional styles, management of interactional breakdowns and enhancement of effective strategies (see Table 2). The results are presented in line with the objectives of the study.

**TABLE 2:** A cross-analysis review of emerging themes in adolescents with autism spectrum disorder’s communication.

Variables	Similarities	Differences
Interactional styles	Verbal communication was the basis of interaction for all four adolescents, with each case experiencing different feelings and difficulties.	Health professionals modelled different non-verbal behaviour (i.e. gestures, visual representation and use of Makaton signs) in the different cases.
Management of interactional difficulties	Repetition when misunderstood was common for the adolescents, parents and health professionals.	Each health professional had a different method that is visual cues, being predictable and direct, using written schedules and talk therapy, depending on adolescents’ needs.
Enhancement of effective strategies	All individuals involved in the cases felt that learning and utilising effective interactional strategies would be beneficial in enhancing communication and future interactions with individuals who have ASD.	Whilst all individuals felt that learning effective interactional strategies would be beneficial, the psychiatrist felt that it may be more pragmatic for therapists as opposed to doctors, as their involvement is largely medical and surrounding correct dosage of medication.

ASD, autism spectrum disorder.

### Ethical considerations

Ethical clearance to conduct this study was obtained from the School of Human & community Development SPPA Human Research Ethics Internal Committee (reference number: No-STA_2018_02) before the study commenced. In adherence, all ethical principles were maintained. Special considerations were made for the adolescents with ASD as they form part of a vulnerable population, hence, assent and participation information forms were adapted, accordingly.

## Results

### Objective 1: To determine the interactional styles and communication breakdown of adolescents with autism spectrum disorder in general life, as stated by the adolescents (and their parents)

Interactional styles and challenges in therapy sessions or consultations were a prevalent feature in all four cases, with some noteworthy similarities and contrasts identified.

All four adolescent participants stated that they employed the approach of talking when dealing with their health professional, despite their diverse feelings and difficulties with communicating. They appeared to use primarily verbal communication during sessions. When the researcher asked P3 the question: ‘do you like talking to the therapist? If so, why, or why not?’ He responded: ‘I like talking.’

There were both similarities and variances in the attitudes of health professionals and parents regarding therapy sessions or consultations. According to their health professionals, P1 and P2 did utilise gestures on occasion**s.** Furthermore, P2 and P4 appeared to articulate single word utterances. Three of the adolescents, notably P2, P3 and P4, appeared to mumble, rendering their speech inaudible at times. In terms of interactional styles, all the health professionals interacted with their adolescent clients primarily through verbal communication.

Several parallels were discovered when examining the interactional issues encountered by these adolescents. The adolescents’ vocabulary was limited, according to the neuro-physiotherapist, and this was also mentioned by P2’s mother and OT. Furthermore, three adolescents displayed poor eye contact, but P1 reacted differently. Initially, P1 struggled with eye contact but as he became more familiar and comfortable in engaging with his therapist, this improved noticeably.

It was also discovered that social functioning and related worry or stress were emphasised for the two teenagers who had been diagnosed with Asperger’s syndrome (P3 and P4). As a result, P3’s mother observed that her son can verbalise, understand and articulate sentences together. She stated:

‘He has problems socially, as well as severe anxiety, which is his greatest hurdle, and this frequently functions as a barrier to interactions’.

Similarly, when P4’s mother was asked about communication challenges her son experiences socially, she stated that:

‘…if he needs to be in a social environment, it makes him very uncomfortable and very stressed’.

The psychiatrist confirmed P4’s mother’s claim by noting that this adolescent lacks the social value of conversing and engaging with others. Within treatment sessions and consultations with these adolescents, different interaction styles and challenges are evident, and these variations are addressed in the within case analysis. This theme was present across all four cases in the across-case analyses and similarities exist.

In conclusion, this shows that parents and healthcare professionals are aware of the adolescents’ unique styles of interactions and are able to respond appropriately. However, the adolescents with ASD, seem to prefer using spoken language despite their challenges with communication. The insistence on spoken language maybe an outcome of having prolonged speech-language intervention.

### Objective 2: To determine the interactional styles and communication breakdown of adolescents with autism spectrum disorder when communicating with their respective health professionals

Interactional challenges were experienced by the adolescents with ASD when communicating with their health professionals. P1, P3 and P4 utilised techniques to deal with interactional challenges, but P2 did not. When P1’s neuro-physiotherapist did not understand him, he kept talking until she did understand. P3 and P4’s strategy for dealing with their inability to understand what their health professional said was to request the health professional to repeat themselves. The strategy of requesting repetition could be a form of asking for clarification, as well as providing time for processing of information. Requesting clarification is an expected strategy use by P3 and P4, as they were on the higher cognitive level of the spectrum.

Similarities and variances in how the health professionals handled interactional challenges that arose during therapy sessions or consultations were observed. The neuro-physiotherapist, OT and psychologist used repetition as a tactic, when requesting information from the adolescents.

The neuro-physiotherapist kept asking the youngster to repeat what he had said, and then she repeated what he had said to make sure that she had heard him correctly. The OT emphasised that she needed to repeat herself frequently, because of the adolescent’s poor attentiveness. Furthermore, the psychologist asked the adolescent to repeat himself, as the adolescent’s speech was mumbled, with little clarity. In addition, the neuro-physiotherapist, OT and psychologist used a similar strategy of shortening phrases and in general, speaking more simply.

For example:

The OT reported: ‘They need to have stuff repeated constantly, else basic communication skills slip away’.

The neuro-physiotherapist: ‘When the adolescent said something ‘I’d say to him’, ‘Okay, is this what you said to me?’ and then repeat it back to him and then he would either correct it or say no’.

Although the neuro-physiotherapist used basic terms, she also had to recommend that the adolescent speak more simply because he wanted to use more advanced language and complex words, which made it difficult to hear and understand him because he was not always able to explain clearly.

It can be concluded that adolescents have some communication repair strategies, such as repetition, however, these strategies are not always successful when there is a breakdown. The communication partners in this study were able to provide support in repair strategies such as asking the adolescents to repeat their messages.

### Objective 3: To determine the interactional styles and communication breakdown experienced by health professionals when communicating with adolescent clients with autism spectrum disorder

The OT and psychologist both acknowledged their use of simple sentences regarding this style. Furthermore, these two health professionals expressed concerns regarding the sensory overload of verbal communication on youngsters, resulting in the use of less speech at times.

According to the father of P1, the health professional frequently explains what will happen in the session and why they are doing various activities, which is part of what the OT also does. In terms of explanations, the health professionals, according to the parents of P1 and P4, provide good explanations to the adolescents in a way that they comprehend. Furthermore, both the health professionals and parents of P2 and P3 explained that these teenagers had trouble grasping and applying complex concepts. P2’s OT stated that his poor concentration had a detrimental impact on his relationships in treatment, which aligns with what P4’s mother stated: ‘he has a tremendous problem with focus’.

The psychiatrist summed up the different approaches by stating:

‘… They don’t always present the same in everybody but I think that is key to understand that and then to understand that individual because everybody is different and I think you can’t have a blanket way of treating everybody with autism’.

In conclusion, the healthcare professionals (HCP) provided repair strategies that aided the adolescents’ comprehension of instructions or messages. Use of simplified instructions aided receptive language of the adolescents.

## Discussion

One of the main features of ASD is impaired verbal and non-verbal communicative capabilities that hinder interactions at home, school, social and therapy environments (Mody & Belliveau 2013). Therefore, the interactional styles and common interactional difficulties demonstrated during sessions between adolescents with ASD and their respective health professionals, impact the success of their treatment. The adolescents with ASD do not always improve as expected in terms of health, rapport between the adolescent and health professional, as well as the feelings and emotions brought on by these interactional difficulties.

This study’s findings raise several challenges concerning future knowledge of HCP interactional styles and applied research efforts with adolescents with ASD. Firstly, the findings imply that investigations of HCP interactional techniques with adolescents with ASD should be planned to take into consideration the effects of the co-verbal interactant’s conduct. The findings of research comparing the communication breakdowns of adolescent or children with ASD with different conversational partners are frequently reported and interpreted in terms of fundamental differences such as age, gender and IQ score in either the children or their partners. However, the teenagers’ interpersonal and communication problems, as well as HCP communication challenges, are rarely considered. The findings of this study imply that the interactional styles and communication breakdowns of adolescents with ASD may be a crucial component influencing the outcomes of such research. The adolescents with ASD who have complex communication difficulties including initiating topics, asking questions and producing topical comments when communicating with their healthcare providers were able to demonstrate reasonable competence in these areas when their healthcarers understood their difficulties.

Secondly, the findings of this research study imply that to effectively understand the communicative breakdowns and interactional styles throughout therapy sessions, the adolescents need to be allowed to reflect and have insights into their communication challenges. According to Larsson et al. (2014), an adolescent’s perspective of his or her communication breakdowns is crucial to success in therapy, as this has implications for the type of intervention. Such analyses and comparison of communication breakdowns and interactional styles during therapy sessions will aid in reducing the inaccuracies and underestimation of the communication needs of adolescents with ASD. Analysis of communication is pivotal as it places a value on the practitioner’s ability to comprehend the adolescent and for the adolescent to accept his or her current ASD outcomes. Health professionals in this study have interacted with their adolescent clients mainly through verbal communication; however, gestures, body language, modelling, physical demonstrations, direct questions and simple guidance were introduced by health professionals as a means of enhancing interactions.

Thirdly, the findings of this study are significant as the research studies have shown that poor communication is associated with poor health outcomes, poor patient compliance, and low patient commitment to intervention and treatment regimens (Levin 2005; Macdonald et al. 2007). A supportive professional interaction style in this study seemed to encourage the adolescent to participate and cooperate positively in therapy. In addition, the professionals were able to intuitively understand what their clients were thinking or feeling, which allowed and encouraged spontaneous comments and use of gestures and other augmentative strategies. The adolescents were then able to formulate and produce a relatively understandable utterance. On the contrary, recent data reveal that it is difficult to intuitively understand what people with ASD are thinking or feeling (Edey et al. 2016).

Communication breakdowns experienced by health professionals ranged from impaired comprehension when communicating to difficulty maintaining the presence and focus of the adolescents with ASD. Therefore, analyses of interactional styles are fundamental to developing strategies that would improve communication and rapport between the adolescents with ASD and their healthcare professionals. Furthermore, effective communication and good rapport will develop confidence in the adolescents with ASD.

## Conclusion

Positive results and significant contribution to knowledge, regarding the interaction styles and communication challenges of adolescents with ASD and their respective health professionals, have been established in this study. The key interactional styles used in therapy sessions or consultations by adolescents with ASD included verbal communication, with some diversity amongst the various cases. However, these interactive styles remain unchanged in all conditions of their lives. Conversely, all health professionals in this study adapted their style for their adolescent clients with ASD. Health professionals therefore used verbal contact to gain interaction and reduce the social anxiety faced by teenagers. Their verbal communication was simplified, more direct and straightforward. It was also clear that there were relationship challenges between health practitioners and adolescents with ASD. For some of these teens, especially those diagnosed with Asperger’s syndrome, anxiety about social interactions tends to greatly hamper interaction skills.

Although the interaction breakdowns varied, most adolescents, parents and health professionals used methods to manage these breakdowns. Not only did health practitioners change their interaction styles, but there was also an eagerness to learn about and establish techniques that would strengthen the experiences they had with adolescents with ASD in a way that would improve the results of therapy sessions and daily life positively. When communicating and engaging with patients with ASD, there is a need for more training that will ensure successful consultation, learning and social results that support not only youth but also health professionals, parents and teachers. Training sessions for healthcare professionals, parents and professionals from cognate fields are essential for improved communication with adolescents with ASD. Speech-language therapists could be part of a team in organising these workshops and training programmes. Collaboration amongst professionals is important in finding effective solutions to mutually experienced communication challenges. The cognitive behavioural therapy (CBT) approach, which began in the mental health community, is steadily gaining support as a viable treatment method for individuals with ASDs who have strong verbal communication abilities, as is the case of individuals with Asperger’s syndrome (Attwood 2012). The CBT provides a concrete method through which social expectations (perceptions, thoughts and emotions) can be discussed and then social-behavioural adaptations, better known as social skills can be defined. Other programmes can also be designed and tested that promote effective pragmatic communication skills.

In addition, recent studies have called for further research on ASD in adolescence and adulthood, as there is a lack of research on autism from teen years to adulthood, especially on knowledge from adolescence to adulthood (Sarris 2013). This study answers this call for ASD research with adolescence. This research study yielded results that inform knowledge on interaction types and coping methods used by adolescents with ASD and their respective health professionals in the South African context.

## References

[CIT0001] Anderson, K., 2013, *Supporting success for children with hearing loss*, viewed 21 April 2018, from https://successforkidswithhearingloss.com 8.

[CIT0002] Attwood, T., 2004, ‘Cognitive behaviour therapy for children and adults with asperger’s syndrome’, *Behaviour Change* 21(3), 147–161. 10.1375/bech.21.3.147.55995

[CIT0003] Baron-Cohen, S., 1995, *Mindblindness: An essay on autism and theory of mind*, The MIT Press, Cambridge, Massachusetts.

[CIT0004] Brady, N.C. & Halle, J.W., 2002, ‘Breakdowns and repairs in conversations between beginning AAC users and their partners’, In J. Reichle, D.R. Beukelman & J.C. Light (eds.) *Exemplary practices for beginning communicators: Implications for AAC*, pp. 323–351, Paul H. Brookes, Baltimore.

[CIT0005] Braun, V. & Clarke, V., 2013, ‘Teaching thematic analysis: Overcoming challenges and developing strategies for effective learning’, *The Psychologist* 26(2), 120–123.

[CIT0006] Buller, M.K. & Buller, D.B., 1987, ‘Physicians’ communication style and patient satisfaction’, *Journal of Health and Social Behavior* 28(4), 375–388. 3429807

[CIT0007] Carter, N., Bryant-Lukosius, D., DiCens, A., Blythe, J. & Neville, A.J., 2014, ‘The use of triangulation in qualitative research’, *Oncology Nursing Forum* 41(5), 545–547. 10.1188/14.ONF.545-547 25158659

[CIT0008] Cesa, C.C. & Mota, H.B., 2017, ‘Augmentative and alternative communication’, *Speech, Language, Hearing Sciences and Education Journal* 19(4), 529–538. 10.1590/1982-021620171943117

[CIT0009] Creswell, J.W. & Creswell, J.D., 2012, *Research design: Qualitative, quantitative, and mixed methods approaches*, 5th edn., Sage, London.

[CIT0010] Edey, R., Cook, J., Brewer, R., Johnson, M.H., Bird, G. & Press, C., 2016, ‘Interaction takes two: Typical adults exhibit mindblindness towards those with autism spectrum disorder’, *Journal of Abnormal Psychology* 125(7), 879–885. 10.1037/abn0000199 27583766

[CIT0011] Halle, J., Brady, N.C. & Drasgow, E., 2004, ‘Enhancing socially adaptive communicative repairs of beginning communicators with disabilities’, *American Journal of Speech-Language Pathology* 13(1), 43–54. 15101813 10.1044/1058-0360(2004/006)

[CIT0012] Howlin, P. & Taylor, J.L., 2015, ‘Addressing the need for high quality research on autism in adulthood’, *Autism* 19(7), 771–773. 10.1177/1362361315595582 26649371

[CIT0013] Keen, D., 2003, ‘Communicative Repair Strategies and Problem Behaviours of Children with Autism’, *International Journal of Disability, Development & Education* 50(1), 53–64.

[CIT0014] Larsson, I., Fridlund, B., Arvidsson, B., Teleman, A. & Bergman, S., 2014, ‘Randomized controlled trial of a nurse-led rheumatology clinic for monitoring biological therapy’, *Journal of Advanced Nursing* 70(1), 164–175. 10.1111/jan.12183 23772698 PMC4285750

[CIT0015] Levey, S., 2019, *Introduction to language development*, Plural Publishing Inc., San Diego, CA.

[CIT0016] Levin, M.E., 2005, ‘The importance of language and culture in paediatric asthma care’, *Current Allergy and Clinical Immunology* 18, 8–12.

[CIT0017] Macdonald, M.E., Carnevale, F.A., & Razack, S., 2007, ‘Understanding what residents want and what residents need: The challenge of cultural training in pediatrics’, *Medical Teacher* 29(5), 444–451. 10.1080/01421590701509639 17885974

[CIT0018] McMillan, J. & Schumacher, S., 2010, *Research in education: Evidence-based inquiry*, 7th edn., Pearson, New York, NY.

[CIT0019] Miller, A., Vernon, T., Wu, V. & Russo, K., 2014, ‘Social skills group intervention for adolescents with autism spectrum disorders: A systematic review’, *Reviewed Journal of Autism Developmental Disorders* 1, 254–265. 10.1007/s40489-014-0017-6

[CIT0020] Milton, D., 2012, ‘On the ontological status of autism: The double empathy problem’, *Disability and Society* 27(6), 883–887. 10.1080/09687599.2012.710008

[CIT0021] Mody, M. & Belliveau, J.W., 2013, ‘Speech and language impairments in autism: Insights from behavior and neuroimaging’, *North American Journal of Medicine & Science* 5(3), 157–161. 10.7156/v5i3p157 24349628 PMC3862077

[CIT0022] Morrison, K.E., DeBrabander, K.M., Jones, D.R., Faso, D.J., Ackerman, R.A. & Sasson, N.J., 2020, ‘Outcomes of real-world social interaction for autistic adults paired with autistic compared to typically developing partners’, *Autism* 24(5), 1067–1080. 10.1177/1362361319892701 31823656

[CIT0023] Murphy, J., Gray, C.M., Van Achterberg, T., Wyke, S. & Cox, S., 2010, ‘The effectiveness of the Talking Mats framework in helping people with dementia to express their views on well-being’, *Sage Journals* 9(4), 454–472. 10.1177/1471301210381776

[CIT0024] Noble, H. & Smith, J., 2015, ‘Issues of validity and reliability in qualitative research’, *Evidence Based Nursing* 18(2), 34–35. 10.1136/eb-2015-102054 25653237

[CIT0025] Patnaik, E., 2013, ‘Reflexivity: Situating the researcher in qualitative research’, *Humanities and Social Science Studies* 2(2), 98–106.

[CIT0026] Sarris, M., 2013, *Autism in the teen years: What to expect, how to help*, viewed 26 March 2018, from https://iancommunity.org/cs/simons_simplex_community/autism_in_teens.

[CIT0027] Scudder, R.R. & Tremain, D.H., 1992, ‘Repair behaviors of children with and without mental retardation’, *Mental Retardation* 30(5), 277–282. 1435281

[CIT0028] Sicile-Kira, C. & Sicile-Kira, J., 2012, *A full life with autism: From learning to forming relationships to achieving independence*, St Martin’s Press, New York, NY.

[CIT0029] Springer, P.E., Van Troon, R., Laughton, B. & Kidd, M., 2013, ‘Characteristics of children with pervasive developmental disorders attending a developmental clinic in the Western Cape Province, South Africa’, *The South African Journal of Child Health* 7(3), 95–99. 10.7196/SAJCH.530

[CIT0030] Thanh, N.C. & Thanh, T.L., 2015, ‘The interconnection between interpretivist paradigm and qualitative methods in education’, *American Journal of Educational Science* 1(2), 24–27, viewed 14 May 2018, from http://www.aiscience.org/journal/ajes.

[CIT0031] Thurm, A. & Swedo, S.E., 2012, ‘The importance of autism research’, *Dialogues in Clinical Neuroscience* 14(3), 219–222. 10.31887/DCNS.2012.14.3/athurm 23226948 PMC3513677

[CIT0032] Trembath, D., Lacono, T., Lyon, K., West, D. & Johnson, H., 2014, ‘Augmentative and alternative communication supports for adults with autism spectrum disorders’, *Autism* 18(8), 891–902. 10.1177/1362361313486204 24104514

[CIT0033] Wetherby, A.M. & Prizant, B.M., 1993, *Communication and symbolic behavior scale manual*, Applied Symbolix, Chicago, IL.

[CIT0034] Zach, L., 2006, ‘Using a multiple-case studies design to investigate the information seeking behaviour of arts administrators’, *Library Trends* 55(1), 4–21. 10.1353/lib.2006.0055

